# Neuroprotection in Glaucoma

**Published:** 2012-01

**Authors:** Naveed Nilforushan

**Affiliations:** Eye Research Center, Rassoul Akram Hospital, Tehran University of Medical Sciences, Tehran, Iran

Glaucoma is a neurodegenerative disorder in which a variety of complicated mechanisms lead to retinal ganglion cell (RGC) apoptosis and death. According to various theories, factors including elevated intraocular pressure (IOP) and vascular dysregulation primarily contribute to the initial insult during glaucomatous atrophy in the form of obstruction of axoplasmic flow within RGC axons, alteration in optic nerve microcirculation at the level of lamina and changes in the laminar glial and connective tissues. Deprivation of neurotrophic factors, such as the brain-derived neurotrophic factor, and release of a large number of neurotoxic agents within the retina, including glutamate, nitric oxide and free radicals, are the consequences of the above-mentioned events.

Currently, IOP reduction is the only proven clinical therapy available for treatment of glaucoma. Nevertheless, signs of progression can be seen in many patients despite well-controlled IOP. In addition, glaucomatous optic neuropathy (GON) has even been observed in patients with “normal” IOP. Therefore, the pathophysiology of glaucoma and particularly normal tension glaucoma is probably beyond IOP alone. Studies on low pressure glaucoma have demonstrated that in most patients higher intraocular pressure is unrelated to poor visual fields.

During the past decade, most studies more focused on discovering neuroprotective treatments along with IOP lowering medications in order to prevent ganglion cell death or even reverse the process of cell death. Neuroprotection aims at blocking primary destructive events affecting RGCs or optic nerve fibers, enhancing RGC or optic nerve fiber survival mechanisms and finally repairing damage occurring during the progressive, secondary stage of the injury.

The idea of using neuroprotective treatment for management of glaucoma arose from the successful results of employing neuroprotective drugs for treatment of certain neurodegenerative disorders of the central nervous system (CNS) such as Alzheimer’s and Parkinson’s disease. Recent studies have elucidated strong links between mechanisms of cell death in Alzheimer’s disease and glaucoma.

Glaucoma is a chronic and slowly progressive disease, therefore assessment of the efficacy of these drugs is a difficult task and requires a long time to be proven. Certain IOP lowering medications, such as brimonidine and betaxolol have been suggested to possess neuroprotective effects. Recently, the low pressure glaucoma study group released the results of comparing brimonidine with timolol with average follow-up of 30 months. This study showed that patients treated with brimonidine were less likely (9%) to experience visual field progression as compared to subjects treated with timolol (39%). IOP reduction was not significantly different between patients assigned to brimonidine or timolol. Although in this study brimonidine, at least indirectly, demonstrated neuroprotective properties, its mechanism of action is not yet fully understood.

There are other promising medical approaches for glaucoma neuroprotection which are briefly discussed below:

*Glutamate antagonists*: Glutamate is one of the major neurotransmitters in the CNS and retina and elevated levels have been reported in glaucoma. This transmitter may facilitate RGC apoptosis by activation of N-methyl D-aspartate receptors (NMDA), resulting in calcium influx into RGCs and disruption of the cell wall. Memantine is an anatagonist of NMDA which had earlier been proven to be effective in Alzheimer’s and Parkinson’s diseases. Although animal studies confirmed that this drug protects injured neural cells from death, phase III clinical trials on glaucoma in humans failed to demonstrate any clinical benefit. The study showed that glaucoma progression was significantly slower in patients receiving higher doses of memantine as compared to patients receiving lower doses of the medication, but there was no significant benefit when compared to patients receiving placebo.

*Neurotrophins*: The brain-derived neurotrophic factor (BDNF) and ciliary derived neurotrophic factor (CNTF) have been found to be decreased in glaucoma. These factors suppress the intrinsic apoptotic process and activate survival signals. Several investigators are now exploring the use of gene transfer methods to deliver neurotrophic factors. Encapsulated cell technology helps to implant cells that have been genetically modified to produce neurotrophic factors.

*Anti inflammatory agents*: Recently researchers have investigated the immune system as a means of providing neuroprotection against neuronal damage. Cop-1, otherwise known as glatiramer acetate, copolymer-1 and copax-1, is a synthetic, random oligopeptide composed of amino acids tyrosine, glutamate, lysine, and alanine. Cop-1 is a Food and Drug Administration (FDA) approved drug for treatment of multiple sclerosis. It is a low affinity antigen and can evoke both active and passive T-cell mediated immune responses at various sites of injury. It has been demonstrated that Cop-1 reduces damage caused by mechanical injury to the optic nerve or intravitreally administered glutamate. Another study reported that vaccination with Cop-1 leads to significant reduction in RGC death in rat models of ocular hypertension.

*Antioxidants*: It is hypothesized that oxidative stress can cause RGC death by damaging the trabecular meshwork, the optic nerve head and the retina. Targets of oxidative stress relevant to the development of GON are most probably the mitochondria. Reduced levels of the antioxidant agent glutathione and increased serum lipid peroxidation products have been identified in patients with primary open angle glaucoma. Antioxidants such as vitamin C, vitamin E and Ginkgo biloba extract have been shown to improve visual fields in patients with glaucoma but there is still no strong evidence or long term studies to confirm this idea. Melatonin (N-acetyl-5-methoxytryptamine) is an indoleamine, secreted by the pineal gland, which exerts antioxidant properties. Melatonin has been shown to neutralize free radicals and activate some antioxidative enzymes; it can therefore be categorized under neuroprotective agents.

*Mitochondrial augmentation*: Mitochondrial dysfunction has been implicated in neuronal apoptosis and shown to play a role in experimental glaucoma. One *in vitro* study provided evidence that mitochondrial dysfunction accompanying RGC death may be induced by glaucoma-related stimuli such as tumor necrosis factor (TNF)-α and hypoxia. The dopaminergic agent, GLC756, has recently been shown to inhibit TNF-α release from activated rat mast cells, suggesting a potential role for glaucoma neuroprotection. Coenzyme Q10 (CoQ10) is a cofactor which plays a crucial role in energy production via the mitochondrial electron transport chain and also has strong antioxidant properties.

*Stem cells*: Several potential applications of stem cells for neuroprotection have been explored. Transplanted mesenchymal stem/stromal (MSC) cells have demonstrated significant neuroprotection in a rat model of glaucoma. These cells can be isolated from a variety of tissues including the adult bone marrow and may have the potential to trans-differentiate into neural cells. However, in order for stem cells to be successful, their use must be coupled with mechanisms by which newly transplanted RGCs can extend their axons to appropriate structures within the brain.

Neuroprotective interventions are promising in the treatment of glaucoma as adjunctive therapy to conventional IOP lowering medications. There are animal studies with promising results in protecting neuronal cells but no longitudinal study in human subjects has yet proven these observations. Better understanding of the pathophysiology and mechanisms of glaucoma can help investigators find new modalities of treatments in the future.
